# Harms and benefits of mammographic screening for breast cancer in Brazil

**DOI:** 10.1371/journal.pone.0297048

**Published:** 2024-01-25

**Authors:** Arn Migowski, Paulo Nadanovsky, Cid Manso de Mello Vianna

**Affiliations:** 1 Professional Master’s Program in Health Technology Assessment, Teaching and Research Coordination, Instituto Nacional de Cardiologia (INC), Ministry of Health, Rio de Janeiro, Brazil; 2 Division of Clinical Research and Technological Development, Research and Innovation Coordination, National Cancer Institute (INCA), Ministry of Health, Rio de Janeiro, Brazil; 3 Instituto de Medicina Social (IMS), Universidade do Estado do Rio de Janeiro (UERJ), Rio de Janeiro, Brazil; 4 Escola Nacional de Saúde Pública (ENSP), FIOCRUZ, Rio de Janeiro, Brazil; Local Health Authority Caserta: Azienda Sanitaria Locale Caserta, ITALY

## Abstract

**Introduction:**

In the absence of evidence on the effect of mammographic screening on overall mortality, comparing the number of deaths avoided with the number of deaths caused by screening would be ideal, but the only existing models of this type adopt a very narrow definition of harms. The objective of the present study was to estimate the number of deaths prevented and induced by various mammography screening protocols in Brazil.

**Methods:**

A simulation study of cohorts of Brazilian women screened, considering various age groups and screening interval protocols, was performed based on life tables. The number of deaths avoided and caused by screening was estimated, as was the absolute risk reduction, the number needed to invite for screening—NNS, the net benefit of screening, and the ratio of “lives saved” to “lives lost”. Nine possible combinations of balances between benefits and harms were performed for each protocol, in addition to other sensitivity analyses.

**Results and conclusions:**

The most efficient protocol was biennial screening from 60 to 69 years of age, with almost three times more deaths avoided than biennial screening from 50 to 59 years of age, with a similar number of deaths avoided by biennial screening from 50 to 69 years of age and with the greatest net benefit. Compared with the best scenario of annual screening from 40 to 49 years of age, the NNS of the protocol with biennial screening from 60 to 69 years of age was three-fold lower. Even in its best scenario, the addition of annual screening from 40 to 49 years of age to biennial screening from 50 to 69 years of age results in a decreased net benefit. However, even in the 50–69 year age group, the estimated reduction in breast cancer mortality for Brazil was half that estimated for the United Kingdom.

## Introduction

In the absence of evidence on the impact of mammographic screening on overall mortality, many attempts to determine the balance between the harms and benefits of screening compare different outcomes, generating a search for an arbitrary and questionable ideal balance value between these outcomes [1]. These comparisons make it difficult for physicians and women to decide on screening because the benefits are usually expressed by a more severe outcome (mortality from breast cancer) and the harms are expressed by intermediate outcomes that are difficult to understand and less severe, such as false-positive results and rate of overdiagnosis [2]. As a result, these intermediate harm outcomes are generally not as valued by women as the possible benefits [3] and can even be interpreted as a benefit [2]. Due to these limitations, models that could compare the number of deaths avoided with the number of deaths caused by screening would be ideal, but the only existing models of this type adopt a very narrow definition of the harms associated with screening [4, 5]. Some authors argue that if all the harms associated with screening are considered, the probable conclusion is that more screened women than unscreened women would die [6, 7]. However, to date, no published study on the balance between the harms and benefits of this intervention has covered all the harms known to be associated with screening and a potential increase in deaths.

In addition, few studies have attempted to estimate risks and benefits in low-and middle-income countries (LMICs) with lower incidences of breast cancer. Modeling studies published to date have used erroneous assumptions about the natural history of the disease and did not consider the existence of lead-time, length-time and overdiagnosis biases in stage changes resulting from screening. These studies also did not include the harms associated with screening, either their impact on the production of premature deaths or its effect on the quality of life of affected women [8–10]. Brazil is the fifth most populous country in the world, and mammographic screening has been considered a public policy priority for more than 15 years and is widely disseminated throughout the country [11].

The aim of the present study is to estimate the harms and benefits of mammographic screening in Brazil in terms of deaths avoided and caused by mammographic screening, calculating the net benefit and the ratio of lives saved to lives lost and comparing different screening protocols.

## Materials and methods

A simulation study was conducted based on life tables, using secondary national-level demographic, breast cancer screening and multiple-cause mortality data, all available without any identifiable information and freely accessible on the website of the Brazilian Ministry of Health, which is responsible for storing data from various official sources of information in the country. The harm and benefit parameters were obtained through systematic literature searches performed by the authors and have already been described in detail elsewhere [11–13]. S1-S3 Tables in S1 File present a summary of the main data sources that were used for all outcomes included in the simulations to evaluate the potentially lethal harms and benefits of mammographic screening. Cohorts screened and unscreened biennially from 50 to 69 years of age and separately from 50 to 59 years and 60 to 69 years of age were modeled. Both the benefits and the harms of mammographic screening were compared with a control group for which it was assumed that these beneficial or harmful effects were not significant, i.e., the Brazilian population of 2012. This group was chosen as a control group because the benefits and main harms of screening only began to emerge more than 5 years after achieving coverage similar to those achieved by screening trials and suggested by the coverage recorded in 2008 in the country [14]. In total, 480 life tables for estimated harms and five for estimated benefits were constructed from data on screening in Brazil.

Cumulative mortality from breast cancer and from other causes in the Brazilian population was calculated based on the construction of life tables [15]. The cumulative risk of death from breast cancer between 55 and 79 years of age was calculated, assuming that mammographic screening initiated at 50 years takes 5 years to have an impact on breast cancer mortality and that this screening effect remains for another 10 years after its interruption at 69 years of age [16]. This approach allowed the estimation of the absolute effect of mammographic screening in Brazil, both in terms of effectiveness in reducing breast cancer mortality, measured by absolute risk reduction (ARR) and number needed to invite to screening (NNS), and in terms of increased mortality from other causes associated with mammographic screening [11–13]. It also enabled the calculation of the net benefit of screening by subtracting the number of deaths potentially caused by screening from the number of potentially preventable deaths with this same intervention as well as the ratio of “lives saved” by screening to “lives lost” as a function of the harms of screening. In the age group of 40 to 49 years, the cumulative mortality considered was mortality from 45 to 59 years, and in the cohort screened from 40 to 69 years of age, the cumulative mortality considered was mortality from 45 to 79 years, according to the same principle that the reduction in mortality would take 5 years to occur and would last for 10 years after the interruption of screening [16].

The following were considered harms associated with mammographic screening compared to the no-screening scenario: 1) deaths from breast cancer induced by ionizing radiation associated with screening mammograms and repeated tests due to inconclusive screening results classified with a Breast Imaging Reporting and Imaging (BI-RADS) score of 0 or due to the diagnostic investigation of false-positive cases; 2) suicide deaths within 12 weeks after overdiagnosis; 3) cardiovascular deaths within 4 weeks after overdiagnosis; 4) surgical mortality associated with excessive mastectomy (overtreatment); 5) deaths due to Stewart-Treves syndrome, resulting from chronic lymphedema as a complication of excessive mastectomy (calculated only for the scenario most unfavorable to screening); and 6) deaths associated with excessive radiotherapy (overtreatment), including increased deaths due to radio-induced breast sarcoma, cardiovascular diseases, lung cancer and esophageal cancer from 5 years after onset to 15 years after the end of screening. All these harms were calculated based on the hypothetical cohorts of 10,000 women screened.

For the sensitivity analysis of the benefits and harms of screening, three different scenarios were estimated: best scenario for screening, base case (most likely scenario based on the best available evidence) and worst scenario for screening. In general, point estimates were used for the base case, and 95% confidence interval estimates were used for the other two scenarios (S2 and S3 Tables in S1 File). For each screening protocol, these three scenarios for harms of screening and three scenarios for benefits were simulated, totaling nine possible combinations of balances between benefits and harms for each protocol. In addition to these nine scenarios for each protocol, other sensitivity analyses were performed for benefits and harms (S2-S5 Tables in S1 File).

The number of deaths from breast cancer induced by ionizing radiation from mammograms was based on modeling studies on the subject already published in both the national [17] and international [4, 5, 18] literature, considering an attendance rate of 80% at screening. For all screening protocols, in the worst scenario, an increase of 10% in the number of deaths due to radiation from diagnostic investigation tests following false-positive screening was added [4]. Additional sensitivity analyses were performed, considering the variation in the attendance rate and radiation dose related to the types of mammography devices used (S4 and S5 Tables in S1 File).

The calculation of the percentage of overdiagnosis was based on randomized clinical trials in which screening was not offered to the control group after the end of the intervention period [19, 20]. The formula used to calculate the percentage of overdiagnosis was the ratio between the numerator given by the difference in the number of cancers in the arm screened with mammography and the number observed in the control group and the denominator given by the cancers detected during screening in the arm with mammography. The period chosen for recording these numbers was from the beginning of the clinical trials until 5 years after the end of those trials, due to the lead-time effect [19, 21], considering the screening frequency and age range. More detailed explanations of the criteria adopted for the choice of overdiagnosis estimates can be found elsewhere [19].

To estimate the number of breast cancer cases that would be detected by screening, a review of the parameters established based on the BI-RADS classification and of all national reports of cancer detection rate (CDR) for mammographic screening published in the literature was performed (S6 Table in S1 File), and data from the national breast cancer information system [22] available on the Ministry of Health website were used. The estimated CDRs by age group are shown in S7 Table in S1 File.

The number of cases of overdiagnosis was obtained by multiplying the percentage of overdiagnosis by the number of cases of breast cancer that would be detected by screening in the cohort of 10,000 Brazilian women screened in the various screening protocols studied.

Mortality rates due to suicide and cardiovascular causes were calculated by detailed age (per year) in the Brazilian population in 2012. Using the mortality data, life tables were constructed for each age group (in years), considering as the initial number only the women who were overdiagnosed in each age group according to the adopted model and using the increase in relative risk (RR) after breast cancer diagnosis [23].

The deaths associated with excessive radiotherapy (overtreatment) were based on the RRs obtained in meta-analyses of clinical trials for the treatment of localized breast cancer [24, 25]. Life tables were then constructed for each cohort of overtreated women for each age group detailed in years. As there is no direct quality evidence regarding the increased risk of cardiovascular disease or lung cancer with the current radiation doses, the increase in RR with the current radiotherapy techniques was estimated for the present study. Data from meta-analyses of clinical trials with older doses were used, and from the total dose for the heart and lungs, the excess RR (ERR) per radiation dose in grays (Gy) was estimated and applied to the modern average doses in the heart and lung [24]. Deaths due to angiosarcoma associated with overtreatment with adjuvant radiotherapy [26–28] were only included in the worst scenario due to uncertainties in the literature about the magnitude of the increased risk of death [24]. For the sensitivity analysis, the highest doses of radiation were used in the worst scenario, and the variation in the duration of the risk increase with radiotherapy was also evaluated with the worst scenario, with persistence of the risk increase for 20 years, following the observations in a meta-analysis of clinical trials [24]. In the best scenario, the increased risk in the model began only in the fifth year after treatment and ended after 15 years.

To calculate the number of excess biopsies, the procedures resulting from false-positive screening and those from the overdiagnosis investigation were summed. The distribution of false-positive results with indications for biopsy according to age range, screening frequency and breast density were obtained from the literature [29]. The attendance rate used was compatible with that observed in clinical trials of mammographic screening and was also used for the calculation of other harms of screening in the simulations.

Our study used only publicly available secondary sources of anonymized data and no ethical committee approval was required.

## Results

Even in the unlikely event that screening from 40 to 49 years was found to be efficacious (best scenario), the NNS would be much higher, with an ARR value almost 10 times lower than in the best scenario for biennial screening from 50 to 69 years (Table 1). The superiority of the absolute benefit of screening in the age group of 60 to 69 years is evident in the number of deaths from breast cancer avoided in the screening, being almost three times higher than that for the age group of 50 to 59 years in the base case (Table 1). The comparison of the results found for screening benefits in Brazil with other results in the literature are described in S8 and S9 Tables in S1 File.

**Table 1:** Effectiveness of mammography screening in Brazil according to the target population and scenario.

Age group	RR	ARR (%)	NNS	Deaths avoided*
40–49 years (best scenario)	0.92	0.034%	2,964	3.37
40–49 years (additional analysis)	0.93	0.030%	3,387	2.95
40–49 years (additional analysis)	0.88	0.051%	1,976	5.06
50–59 years (base case)	0.86	0.083%	1,205	8.30
50–59 years (best scenario)	0.68	0.190%	527	18.97
50–59 years (worst scenario)	0.97	0.018%	5,623	1.78
60–69 years (base case)	0.67	0.230%	435	22.98
60–69 years (best scenario)	0.54	0.320%	312	32.03
60–69 years (worst scenario)	0.83	0.118%	845	11.84
50–69 years (base case)	0.78	0.229%	437	22.86
50–69 years (best scenario)	0.69	0.322%	310	32.22
50–69 years (worst scenario)	0.90	0.104%	962	10.39
50–69 years (additional analysis)	0.80	0.208%	481	20.78

RR = Relative Risk; ARR = Absolute Risk Reduction

NNS = Number Needed to invite for Screening to avoid a death from breast cancer.

*Deaths avoided in 10,000 women invited to screening in the age group.

With regard to harms, this situation is reversed. The annual screening in the age group of 40 to 49 years approximately doubles the harms, such as false-positive results and excess biopsies, when compared to the biennial screening in the age group of 50 to 59 years or of 60 to 69 years, in addition to generating more cases of overdiagnosis and overtreatment (S10 and S11 Tables in S1 File). It also has the worst ratio between deaths avoided and those caused by screening, even in the best scenario (Table 2). If the harms from the worst scenario are considered, even with the benefits of the best scenario, the net benefit of the annual screening from 40 to 49 years would be only 1.8. In this protocol, the RR of the reduction in breast cancer mortality with screening has to be 0.962 or lower for the benefits to outweigh the harms predicted in the worst scenario in terms of deaths caused by screening.

**Table 2:** Deaths avoided and deaths caused by mammographic screening in Brazil among 10,000 women invited to screen, compared with those without mammographic screening in the respective age groups.

Benefits and harms associated with mammography screening	Number of deaths prevented and deaths caused by mammographic screening								
	Biennial screening from 50 to 59 years old			Biennial screening from 60 to 69 years old			Annual screening from 40 to 49 years old		
	BS	BC	WS	BS	BC	WS	BS	BC	WS
**Breast cancer deaths prevented by screening**	**18.968**	**8.299**	**1.778**	**32.026**	**22.975**	**11.836**	**3.374**	**0.000**	**0.000**
NNS (to prevent a breast cancer death)	527	1205	5623	312	435	845	2964	NA	NA
Deaths from breast cancer radioinduced by mammograms	0.083	0.129	0.141	0.037	0.057	0.063	0.544	0.842	0.926
Deaths caused by overtreatment with surgery	0.000	0.040	0.050	0.000	0.061	0.075	0.000	0.071	0.088
Deaths from cardiovascular disease caused by RT overtreatment	0.103	0.257	0.428	0.673	1.558	2.596	0.073	0.197	0.328
Deaths from lung cancer caused by RT overtreatment	0.075	0.075	0.136	0.151	0.151	0.273	0.081	0.081	0.147
Esophageal cancer deaths caused by RT overtreatment	0.000	0.000	0.028	0.000	0.000	0.073	0.000	0.000	0.027
Deaths from pulmonary embolism caused by RT overtreatment	0.000	0.000	0.023	0.000	0.000	0.089	0.000	0.000	0.020
Deaths from sarcoma caused by RT overtreatment	0.000	0.000	0.018	0.000	0.000	0.027	0.000	0.000	0.032
Suicide deaths associated with overdiagnosis	0.000	0.001	0.002	0.000	0.002	0.004	0.000	0.001	0.004
Cardiovascular deaths associated with overdiagnosis	0.001	0.003	0.003	0.006	0.023	0.023	0.001	0.002	0.002
**Total deaths caused by screening**	**0.262**	**0.505**	**0.830**	**0.867**	**1.851**	**3.223**	**0.698**	**1.194**	**1.574**
**Net benefit from screening** *	**18.706**	**7.793**	**0.948**	**31.159**	**21.124**	**8.613**	**2.676**	**none**	**none**
**Ratio of lives saved to lives lost**	**72.265**	**16.421**	**2.142**	**36.932**	**12.410**	**3.672**	**4.832**	**-**	**-**

RT = Radiotherapy; BS = Best Scenario; BC = Base Case; WS = Worst Scenario

*Number of deaths prevented minus number of deaths caused by screening.

The lowest number of deaths caused by screening occurred with biennial screening from 50 to 59 years (Table 2). This causes the ratio between deaths avoided and deaths caused by screening to be higher in the range of 50 to 59 years than in the range of 60 to 69 years (16.42 versus 12.41) (Table 2). Nevertheless, the net benefit of screening in absolute terms (avoided deaths minus deaths caused by screening) was much higher in the 60-69-year-old age group than in the 50-59-year-old age group (Table 2).

Of all the screening protocols evaluated, the most efficient was biennial screening from 60 to 69 years (Table 2). With only five screening rounds in 10 years, the protocol avoids a similar number of deaths as biennial screening from 50 to 69 years (Table 3) and annual screening from 40 to 49 years followed by biennial screening from 50 to 69 years (Table 3). The ratio between deaths avoided and caused by screening and the net benefit are also similar for biennial screening from 60 to 69 years and biennial screening from 50 to 69 (Tables 2 and 3). The NNS of the biennial screening from 50 to 69 was 310, 437 and 962 for the best scenario, base case and worst scenario, respectively.

**Table 3:** Deaths avoided and deaths caused by mammographic screening in Brazil, based on the screening protocol for 10,000 women invited to screen, compared with those without mammographic screening in the respective age groups.

Benefits and harms associated with mammography screening	Biennial screening from 50 to 69 years old			Annual screening from 40 to 49 years, followed by biennial screening from 50 to 69 years		

	BS	BC	WS	BS	BC	WS
**Breast cancer deaths prevented by screening**	**32.216**	**22.863**	**10.392**	**34.806**	**22.307**	**10.139**
Deaths from breast cancer radioinduced by mammograms	0.120	0.186	0.204	0.664	1.027	1.130
Deaths caused by overtreatment with surgery	0.000	0.101	0.124	0.000	0.172	0.212
Deaths from cardiovascular disease caused by RT overtreatment	0.492	1.142	1.903	0.546	1.293	2.072
Deaths from lung cancer caused by RT overtreatment	0.213	0.213	0.383	0.282	0.282	0.509
Esophageal cancer deaths caused by RT overtreatment	0.000	0.000	0.097	0.000	0.000	0.120
Deaths from pulmonary embolism caused by RT overtreatment	0.000	0.000	0.107	0.000	0.000	0.123
Deaths from sarcoma caused by RT overtreatment	0.000	0.000	0.045	0.000	0.000	0.077
Suicide deaths associated with overdiagnosis	0.000	0.002	0.005	0.000	0.003	0.008
Cardiovascular deaths associated with overdiagnosis	0.004	0.016	0.016	0.004	0.014	0.014
**Total deaths caused by screening**	**0.829**	**1.659**	**2.885**	**1.496**	**2.791**	**4.265**
Net benefit from screening* **(in number of deaths prevented)**	**31.387**	**21.204**	**7.507**	**33.310**	**19.515**	**5.875**
**Ratio of lives saved to lives lost**	**38.850**	**13.779**	**3.602**	**23.272**	**7.992**	**2.377**

RT = Radiotherapy; BS = Best Scenario; BC = Base Case; WS = Worst Scenario

*Number of deaths prevented minus number of deaths caused by screening.

For annual screening in the age group of 40 to 49 years, cancers induced by mammograms were the leading cause of death in all three scenarios (Table 4). S12 Table in S1 File shows the same information but for the cohorts subjected to annual screening from 40 to 49 years, followed by biennial screening from 50 to 69 years. S4 and S5 Tables in S1 File show additional analyses for the number of deaths from breast cancer induced by mammograms.

**Table 4:** Distribution of causes of death (%) associated with mammographic screening.

Cause of death	Annual screening from 40–49 years			Biennial screening from 50–69 years		
	BS	BC	WS	BS	BC	WS
Breast cancer radioinduced by mammograms	77.92	70.46	58.83	14.47	11.19	7.08
Overtreatment with surgery	0	5.97	5.58	0	6.09	4.31
Cardiovascular diseases related to RT overtreatment	10.40	16.49	20.86	59.36	68.81	65.96
Lung cancer related to RT overtreatment	11.57	6.76	9.31	25.65	12.82	13.29
Esophageal cancer related to RT overtreatment	0	0	1.72	0	0	3.37
Pulmonary embolism related to RT overtreatment	0	0	1.29	0	0	3.70
Sarcomas related to RT overtreatment	0	0	2.03	0	0	1.57
Suicide related to overdiagnosis	0.03	0.12	0.23	0.03	0.11	0.16
Cardiovascular diseases related to overdiagnosis	0.08	0.20	0.15	0.49	0.98	0.57
Total	100	100	100	100	100	100

RT = Radiotherapy; BS = Best Scenario; BC = Base Case; WS = Worst Scenario

For biennial screening in the age group of 50 to 69 years, deaths from overtreatment with radiotherapy were the main cause of death in all three scenarios (Table 4, S13 and S14 Tables in S1 File). By dividing the deaths caused by screening into groups of diseases, the first place was occupied by cardiovascular diseases in all scenarios evaluated (59.85%, 69.79% and 70.22%, respectively, in the best scenario, base case and worst scenario), with secondary cancers placing second (Table 4). S15 Table in S1 File shows detailed results for mortality associated with surgery.

## Discussion

To the best of our knowledge, this is the broadest study on harm estimation of breast cancer screening ever published, evaluating the absolute impact of mammographic screening not only in terms of deaths avoided but also considering a broad spectrum of deaths caused by screening. It is common, for example, for deaths from breast cancer induced by radiation from screening mammograms to be considered the only cause of death attributed to screening, in most cases excluding even ionizing radiation associated with false-positive cases, assuming in a biased manner that the benefits outweigh the harms [4, 17, 18, 30]. Even other models that consider other types of screening risks [7, 16] are less comprehensive than the present study in terms of harms of screening. The same occurs in systematic reviews and clinical guidelines [11, 20, 31, 32].

In LMICs, breast cancer incidence and mortality rates are generally lower than the rates in high-income countries (HICs) where all mammographic clinical trials and modeling studies have been performed. This lower incidence and mortality result in lower effectiveness of mammographic screening in LMICs, which currently account for approximately 60% of breast cancer deaths worldwide [33]. This implies a lower absolute effect of mammographic screening, even assuming that the relative effect remains the same as in clinical trials. Nevertheless, in general, clinical screening guidelines developed in HICs have been uncritically adopted by the rest of the world [2, 11].

Several published models on the cost-effectiveness of mammographic screening ignore the harms associated with this intervention [8–10, 34]. They assume that the change in the stage distribution translates into changes in survival, without considering that most of this change is due to lead-time and overdiagnosis biases [11]. An indication of the overestimation of survival gains in these modeling studies is that it is difficult to prove an increase in survival with screening based on data from randomized clinical trials [35].

Another error found, for example, in the model used by the latest guidelines of the American Cancer Society [36], is the use of breast cancer incidence rates to determine the target age range for screening. As screening increases the number of detected (diagnosed) cases, guidelines such as those of the American Cancer Society indicate screening based on this criterion, without considering that many of the cases detected are overdiagnosed. As the screening of young women is widespread in the USA—and in Brazil—the overdiagnosis resulting from this practice ends up becoming an argument for its recommendation, generating a vicious cycle that perpetuates wrong practices and generates excess harms. Conversely, the mortality outcome used in the present model is not influenced by overdiagnosis.

The calculation of the absolute benefit of screening in Brazil revealed an impact similar to that observed in clinical screening trials [37] but considerably lower than the absolute benefit reported in the literature in models created for HICs, even with the use of the same RR reductions. One explanation is that, according to our results, the percentage of Brazilian women between 55 and 79 years who die from breast cancer in a scenario with low mammographic screening coverage is 1.04%. For the United Kingdom, for instance, this value is 2.13%, and it is expected that if 10,000 Brazilian women are invited for biennial mammographic screening for 20 years, 21 deaths from breast cancer would be avoided, not 43 as predicted for the United Kingdom, with an NNS of 481 in Brazil rather than 235 [16]. Therefore, even in this age group, the estimated effect for Brazil in reducing breast cancer mortality was half that estimated for the United Kingdom by the UK Independent Panel, even assuming the same RR and using similar methods. In a model developed for the USA, the estimated number of deaths from breast cancer avoided with biennial screening from 50 to 74 years was three times higher than estimated in the present study: 63 per 10,000 women [4].

The practical implication of these results is that the absolute benefit of mammographic screening in Brazil is lower than that in the UK and other countries with higher breast cancer mortality rates. Our results also apply to countries with lower breast cancer mortality rates in age groups that would benefit from screening. Even assuming the same relative risk reduction observed in clinical trials carried out in Europe and North America, we will have smaller absolute risk reductions in Brazil and in other countries with lower breast cancer mortality. Absolute risk reduction has the most practical importance since it translates into the number of lives actually saved by screening. These population risks of developing breast cancer are dynamic and have increased in Asian and Latin American countries, due to the adoption of a westernized diet and lifestyle, increased prevalence of obesity, lower birth rates, older age at first pregnancy, shorter breastfeeding time, and other factors [38]. These risk factors increase the baseline breast cancer risk of the population in terms of incidence and mortality rates and tends to make screening more effective. However, analyses of recent incidence trends in young women and projections for the coming decades do not indicate a relevant scenario changes in Brazil [39].

The estimation of screening effectiveness in Brazil is further reduced when lower RRs are used in the sensitivity analysis. Estimating a 15% relative reduction in breast cancer mortality suggested by the Cochrane Review [20], for example, with screening from 50 to 69 years, we would have a 0.16% absolute reduction in cumulative mortality from 55 to 79 years in Brazil, an NNS of 641, with 16 deaths from breast cancer avoided. However, it should be considered that this proposal for RR by the Cochrane review refers to clinical trials of mammographic screening in general, in which women aged 40 to 49 years were the most studied group, which explains why the ARR is more compatible with those estimated for the age group of 40 to 49 years in the present study (0.05%). In another systematic review, it was estimated that with biennial or triennial screening, to avoid death from breast cancer, it would be necessary to screen 2,108 and 721 women in the age groups 40 to 49 and 50 to 69 years, respectively [31]. A modeling study suggests that the balance between harms and benefits of triennial screening would be more favorable for low-risk women than that for biennial screening [40], but there is no direct high-quality evidence that demonstrates the superiority of triennial screening [12].

For annual screening from 40 to 49 years, even in an optimistic scenario, where it is considered effective, the balance between prevented and caused deaths would be borderline, and the effectiveness would be much lower, with an NNS of approximately triple the value estimated for the biennial screening from 60 to 69 years and a number of deaths avoided seven-fold lower than that in this age group. If the current recommendation of biennial screening from 50 to 69 years was changed by advancing the beginning of screening to 40 years of age, with an annual frequency until 49 years of age, even if considered to be effective, the net benefit of screening would decrease when compared to the currently recommended protocol in the country [12], if considering the most likely scenario of harms from screening. The use of overestimated relative risks reductions of mortality with screening and lack of information on absolute risks remain key problems even in the most recent modeling studies that recommend screening in women under 50 years of age [41].

The guidelines of the American College of Physicians agree with the results presented here: in women between 40 and 49 years of age, the potential harms of screening outweigh its benefits, and in addition to lower absolute benefits, a larger number of false positives and a greater degree of overdiagnosis occur in this age group [42]. The alleged transient reduction in mortality in the first 10 years of follow-up [43] may be due to the “left-to-nature” design of the UK Age Trial, which may have improved awareness and medical care in the intervention group and may have influenced the determination of the cause of death [44].

Although competing causes of death are more common in elderly women, the risk of breast cancer is also greater in this group than it is in younger women, contributing to a greater reduction in the absolute risk of death from breast cancer with mammographic screening. Therefore, although we have used real mortality rates in the country by age group and life tables, both considering competing causes of mortality, this did not compromise the effectiveness of screening in the older group. On the other hand, regarding the risks of screening in the age group of 60–69 years, competing causes of mortality influenced our results, as older women have a higher baseline risk of cardiovascular disease, which increases with overtreatment of breast cancer. Even so, the net benefit was the most favorable in this age group. However, it is also important to consider the age to stop screening; in women ≥ 70 years, there is insufficient evidence of screening benefit and an increased risk of overdiagnosis [45]. Moreover, if we were to include screening among women over 70 years of age, there could be a reduction in the estimated absolute benefit due to comorbidities.

The number of deaths due to cancer induced by screening mammograms used in the present study was consistent with other studies in the literature. For example, the number of deaths in the age group of 50 to 59 years for Brazil was identical to the number of deaths estimated for the age group of 50 to 59 years in the Canadian model [30]. However, the numbers used for Brazil show at least one source of underestimation: radiation from additional tests resulting from false-positive results was not added to the model. Another factor that may have contributed to the underestimation is that the Brazilian study estimated mortality and incidence up to 85 years, while other studies estimated it for life or up to 100 years [4].

Women with very large breasts need a higher dose of radiation per examination and a higher number of incidences, resulting in an excess of 21 deaths per 100,000 [4]. This situation is similar to that of women with breast implants, which are very prevalent in Brazil. Thus, the simple consideration of this increased risk associated with breast volume would eliminate any possible net benefit of annual mammographic screening from 40 to 49 years even in the most optimistic scenario used in the present study. This is without considering that obese women are 20% more likely to have false-positive results than are normal-weight women [4].

False-positive results were not included in the harm-benefit simulations presented herein due to the lack of impact on increased mortality. Despite this, false-positive results are the most common harms of mammographic screening, and the negative psychological effects associated with them can last up to three years [46]. The parameters adopted in the present study for the estimation of false-positive numbers [29] are in agreement with another population-based study conducted in the USA, both with regard to the cumulative percentage of false positives in general and women referred for biopsy [47]. In the present study, the false-positive probability parameters used were those from the USA, a country that has opportunistic screening and referral criteria similar to those used in Brazil.

Despite the difference in invasiveness, morbidity and complications, especially with regard to surgical biopsy, no study to date has reported deaths directly related to any of these types of breast biopsies, including a series of 55,936 biopsies evaluated [48]. As in the present study in which the estimates were made for 10,000 women, it is unlikely that the excess biopsies associated with screening could have any impact on increasing mortality. In addition, the harms associated with biopsies are not described in the screening guidelines [42].

For biennial screening from 50 to 69 years, we estimated that the largest number of deaths caused by screening occurs due to cardiovascular deaths associated with overtreatment with adjuvant radiotherapy, accounting for approximately 70% of the total deaths caused by screening in the most likely scenario, even when considering the modern radiation doses [24]. The new deep inspiration breath hold technique can decrease the radiation dose to the heart, both in intensity modulated radiation therapy (IMRT) and in volumetric modulated arc radiotherapy (VMAT). The second main probable cause of death in this group was lung cancer, which was also associated with overtreatment with adjuvant radiotherapy, corresponding to 13% of all deaths caused by screening. One factor that can simultaneously increase the absolute risks of these two complications is current smoking by women with a high smoking load (pack-years). However, this increased risk can be mitigated by smoking cessation, even when it occurs at the time of radiotherapy. Another encouraging prospect for the future reduction of these harms in older women are the recent results of PRIME II trial, which showed that omission of adjuvant radiotherapy did not affect survival in women 65 years or older with T1-T2, node-negative, estrogen receptor positive breast cancer [49].

### Other factors influencing the overdiagnosis estimates

Even the most recent modeling studies fail to estimate overdiagnosis, due to overestimation of the sojourn time and lack of estimation of LMICs with lower life expectancies [50].

We chose to use the total number of cancers detected by screening mammography in 5 years of follow-up as the denominator because larger denominators, such as the total number of cancers detected in the experimental groups in long follow-up periods after studies have ended, greatly dilute the estimates of overdiagnosis [19, 51].

We estimated 102 cases of overdiagnosis for the cohort of screened individuals aged 50 to 69 years, while the estimate by the independent panel in the United Kingdom was 129 cases [16]. One of the factors that determined this difference was the higher CDR used in the calculations for the United Kingdom, estimated from data from the National Health Service (NHS) screening program.

The CDR increases with age and decreases in subsequent screening rounds because first-time examinations capture prevalent cases. With the NHS data cited by the independent panel, it is possible to infer that the mean CDR used was 0.80%, without considering interval cancers [16]. In the USA, the CDR for screening is 0.51%, based on data from the Breast Cancer Screening Consortium [52, 53]. As the CDR estimated here was low and was used only to estimate cases of overdiagnosis, it may have underestimated the harms caused by screening in Brazil.

We used screening adherence (attendance rate) to reduce the estimates for cases of overdiagnosis, thus seeking to use the same intention-to-treat logic used to calculate the RRs of mortality reduction in clinical trials. Thus, we seek to balance the estimates of benefits and harms. The attendance rate used was the same as that used by the independent panel of the United Kingdom, which in turn was based on the attendance rates observed in most clinical trials of mammographic screening [16, 20]. In a program with active participation of women in southern Brazil, adherence to biennial screening was 80%, exactly the estimate used in the present study [54].

### Limitations and future directions

In all scenarios, it was considered that the reduction in RRs in Brazil today would be similar to those observed in clinical screening trials. This assumption was also adopted in the main published models [16, 31, 37]. A criticism that could be made to this approach is that, in theory, the evolution of adjuvant therapy in recent decades may have reduced the RR of screening because it improved the prognosis of locally advanced tumors, decreasing the contrast with the control group [55].

Despite this rationale, estimating the real impact of adjuvant therapy in modifying the RRs of screening is an extremely difficult task, to the point that some authors consider that the only solution would be to start a new mammographic screening trial [56]. However, it would take approximately 20 years to complete a new study, and the therapies used during the study would probably be outdated by the end. Given this impasse, some authors have tried to estimate this effect of adjuvant therapy on the screening effect. One of the leaders of adjuvant therapy clinical trials proposed that using 5-year survival in a clinical trial of adjuvant hormone therapy (90%) and based on the incidence of breast cancer and maintaining an RR of 0.8 with screening, the number of deaths avoided in the UK should be four deaths, not 43, as proposed by the independent panel [7]. However, the use of survival as an outcome overestimates the effect of the evolution of adjuvant therapy because mammographic screening has increased in prevalence during this period, and the lead time and cases of overdiagnosis spuriously increase the survival time. Conversely, a modeling study created a hypothetical screening trial in 2015 and compared it with a 1975 clinical trial, concluding that there was no decrease in RRs with screening across time [56].

A possible future direction for the present study would be to stratify the models by more variables that influence screening effectiveness. Although the international scientific community is increasingly interested in the notion of individualizing screening decisions, there are currently no validated guidelines in the literature that recommend adaptations according to the personal risk of death from breast cancer. Generally, only sex and age are used to define screening recommendations, with the only exception occurring in very high risk individuals. Although promising for reducing harms and maximizing benefits [15], risk-based screening still requires more evidence to establish risk factors, validate predictive models and implement them in clinical practice [57], as well as more data on feasibility and acceptance by the target population [58]. It would also be interesting to include factors that increase the harms of screening, such as having large breasts, which increases the risk of cancer induced by mammograms, or smoking, which increases the risk of the main long-term complications of overtreatment with radiotherapy, such as coronary artery disease and lung cancer.

One of the main bases for estimating the harms and benefits of mammographic screening in the present study was the Brazilian mortality data from the Mortality Information System. Implemented in the country approximately 45 years ago, this system covers the entire national territory and practically 100% of the deaths that occur in the country. In an evaluation conducted in the early 2020s, Brazilian mortality data were classified in the same quality category as those of European countries such as Germany, France, Italy, Spain, Switzerland, Sweden, Norway, Denmark, Belgium and the Netherlands [59]. Due to improvements in the quality of death records in Brazil in the last two decades, there has been not only a significant increase in the coverage of mortality records [60] but also a gradual decrease in the percentage of ill-defined causes [61]. For cancer, the impact of mortality from ill-defined causes tends to be even lower. With the use of a reclassification coefficient of ill-defined causes of death, based on the investigation of deaths in Brazil, cancer is among the group of diseases with the lowest increase in deaths after reclassification [60].

The decision not to include excess mortality from ipsilateral and contralateral breast cancer induced by overtreatment with radiotherapy was adopted due to a lack of data in the literature. This choice probably underestimates, to some extent, the harms of overtreatment because the excess incidence and late mortality from breast cancer would not be accounted for in the calculations of treatment efficacy in clinical trials.

One of the main limitations of the present study is the lack of inclusion in the analyses of the temporality of deaths. Deaths closer to the date of diagnosis of breast cancer detected by screening have a greater impact on actual survival, as is the case for surgical deaths due to excessive mastectomies and cardiovascular complications and suicide deaths that occur shortly after the overdiagnosis of breast cancer. Regarding suicide deaths, recent evidence confirms the increased risk those with situ/localized cancer in general and specifically with breast cancer not only in the short term, as in our article, but also two years after diagnosis [62], while another recent study found a higher incidence of suicide between 6 months and one year after diagnosis [63]. However, these cases represent the minority of all deaths caused by screening. Both, deaths potentially prevented by screening, and most deaths caused by screening, tend to occur between 5 and 15 years after screening. Thus, there seems to be some balance regarding the temporality of deaths caused and prevented by screening. One way to introduce the dimension of lifetime in the analyses would be to calculate the potential years of life lost. Considering the probable lack of effectiveness in the screening of women aged 40 to 49 years, the inclusion of years of life lost in the analyses would further increase the negative impact of the harms of this practice. For example, in a model of cancer induced by ionizing radiation, the number of years of life potentially lost from deaths due to cancer induced by screening mammograms was more than six-fold higher for annual screening from 40 to 49 years than for biennial screening from 50 to 59 years [30].

Importantly, the real scenario in Brazil may be even more unfavorable than those presented in this study. The modern radiation doses used in the simulations of harms from overtreatment with adjuvant radiotherapy are not doses from Brazilian studies because there are no data available. The surgical mortality data are also not Brazilian data for the same reason. The same occurs with the RR of reduced mortality, used to estimate the absolute benefit of screening, which was derived from clinical trials. These RRs may be overestimated for Brazil and other LMICs, as the estimates assume the quality assurance of mammograms and access to the entire line of care, including timely diagnostic confirmation and treatment.

Another limitation of the present study is the non-inclusion of outcomes related to quality of life, given the recognized psychological effects of common screening outcomes such as false-positive results [46] and the overdiagnosis of breast cancer [23]. Although deaths by suicide associated with overdiagnosis were considered in the study, less extreme cases, such as depression, anxiety and posttraumatic stress disorder, are more prevalent and may be persistent [64]. Non-lethal adverse effects of overtreatment were also not considered in the present study, such as radiotherapy-induced acute dermatitis, which is common but generally of moderate intensity [65, 66]. It is also necessary to consider that the increased risk of cardiovascular diseases after overtreatment with radiotherapy, for example, was only evaluated in terms of mortality, but certainly its increased incidence also has repercussions in terms of quality of life.

## Conclusion

The present study indicates that the benefits of mammographic screening in terms of deaths avoided by screening outweigh its harms in terms of deaths caused by screening in biennial screening from 50 to 59 years, 60 to 69 years and 50 to 69 years. None of the sensitivity analyses altered this conclusion for these screening protocols. However, even in this age group, the estimated effect for Brazil in reducing breast cancer mortality was half that estimated for the United Kingdom, for instance, even assuming the same RR. Although false-positives and cases of overdiagnosis are more frequent than both deaths caused and prevented by screening, our results offer an additional and more direct comparision between benefits and harms.

Comparing all the screening protocols analyzed, the most efficient is biennial screening from 60 to 69 years, with almost three-fold more deaths avoided than for biennial screening from 50 to 59 years and with a similar number of deaths avoided as biennial screening from 50 to 69 years.

Screening from 60 to 69 years also has the greatest net benefit. Compared with the best scenario for annual screening form 40 to 49 years, the NNS of biennial screening from 60 to 69 years is three-fold lower. Even in the best scenario, the addition of annual screening from 40 to 49 years to biennial screening from 50 to 69 years results in a decreased net benefit of screening. Therefore, screening in the age group of 60 to 69 years should be prioritized, which contrasts with the reality of media campaigns on screening that are generally aimed at young women.

To reduce the harms caused by mammographic screening in Brazil and to ensure that the balance between harms and benefits is likely to be favorable, the main measures that should be adopted are as follows: incentives for adherence to biennial screening and only in the target age group of 50 to 69 years (and especially 60 to 69 years), discouraging screening outside this protocol; informed and shared decision-making before a referral for screening, especially when it is requested outside of this age range, which may rely on decision aids based on the results of the present study alongside the already traditional numbers of false-positives and overdiagnosis cases; the implementation of national mammography and radiotherapy quality programs; cessation of smoking by patients undergoing adjuvant radiotherapy; and the adoption of new protocols omitting radiotherapy in older women with low risk breast cancer.

## Supporting information

S1 File (DOCX)

## References

[1] World Health Organization. WHO position paper on mammography screening. Geneva, 2014. Available at: https://apps.who.int/iris/bitstream/handle/10665/137339/9789241507936_eng.pdf;jsessionid=21ADC13AF7578AB75DD008909FE6195E?sequence=1 (Accessed on September 26, 2023). 25642524

[2] Migowski A, Dias MBK, Nadanovsky P, Silva GAE, Sant’Ana DR, Stein AT. Guidelines for early detection of breast cancer in Brazil. III—Challenges for implementation. Cad Saude Publica. 2018 Jun 25;34(6):e00046317. English, Portuguese. doi: 10.1590/0102-311X00046317 29952397

[3] Qin X, Nagler RH, Fowler EF, Gollust SE. U.S. women’s perceived importance of the harms and benefits of mammograms and associations with screening ambivalence: Results from a national survey. Prev Med. 2019 Jun;123:130–137. doi: 10.1016/j.ypmed.2019.03.023 30890352

[4] Miglioretti DL, Lange J, van den Broek JJ, Lee CI, van Ravesteyn NT, Ritley D, et al. Radiation-Induced Breast Cancer Incidence and Mortality From Digital Mammography Screening: A Modeling Study. Ann Intern Med. 2016;164(4):205–14. doi: 10.7326/M15-1241 26756460 PMC4878445

[5] Berrington de González A; Reeves G. Mammographic screening before age 50 years in the UK: comparison of the radiation risks with the mortality benefits. Br J Cancer. 2005 Sep 5;93(5):590–6. doi: 10.1038/sj.bjc.6602683 16136033 PMC2361593

[6] Gøtzsche PC. Mammography screening is harmful and should be abandoned. J R Soc Med. 2015 Sep;108(9):341–5. doi: 10.1177/0141076815602452 26359135 PMC4582264

[7] Baum M. Harms from breast cancer screening outweigh benefits if death caused by treatment is included. BMJ. 2013 Jan 23;346:f385. doi: 10.1136/bmj.f385 23344314

[8] Ribeiro RA, Caleffi M, Polanczyk CA. Custo-efetividade de um programa de rastreamento organizado de câncer de mama no Sul do Brasil [Cost-effectiveness of an organized breast cancer screening program in Southern Brazil]. Cad Saude Publica. 2013 Nov;29 Suppl 1:S131–45. Portuguese. doi: 10.1590/0102-311x00005213 25402242

[9] Zelle SG, Vidaurre T, Abugattas JE, Manrique JE, Sarria G, Jeronimo J, et al. Cost-effectiveness analysis of breast cancer control interventions in Peru. PLoS One. 2013 Dec 10;8(12):e82575. doi: 10.1371/journal.pone.0082575 ECollection 2013. 24349314 PMC3859673

[10] Zelle SG, Nyarko KM, Bosu WK, Aikins M, Niëns LM, et al. Costs, effects and cost-effectiveness of breast cancer control in Ghana. Trop Med Int Health. 2012 Aug;17(8):1031–43. doi: 10.1111/j.1365-3156.2012.03021.x 22809238

[11] Migowski A, Stein AT, Ferreira CBT, Ferreira DMTP, Nadanovsky P. Guidelines for early detection of breast cancer in Brazil. I—Development methods. Cad Saude Publica. 2018 Jun 21;34(6):e00116317. English, Portuguese. doi: 10.1590/0102-311X00116317 29947660

[12] Migowski A, Silva GAE, Dias MBK, Diz MDPE, Sant’Ana DR, Nadanovsky P. Guidelines for early detection of breast cancer in Brazil. II—New national recommendations, main evidence, and controversies. Cad Saude Publica. 2018 Jun 21;34(6):e00074817. English, Portuguese. doi: 10.1590/0102-311X00074817 29947654

[13] Migowski A, Nadanovsky P, Vianna CMM. Cardiovascular Mortality Associated with Mammographic Screening. Revista Brasileira de Cancerologia 2019 Oct; 65(3): e–02335. doi: 10.32635/2176-9745.RBC.2019v65n3.335

[14] Barbosa YC, Oliveira AGC, Rabêlo PPC, Silva FS, Santos AMD. Factors associated with lack of mammography: National Health Survey, 2013. Rev Bras Epidemiol. 2019 Dec 5;22:e190069. Portuguese, English. doi: 10.1590/1980-549720190069 31826123

[15] Pashayan N, Morris S, Gilbert FJ, Pharoah PDP. Cost-effectiveness and Benefit-to-Harm Ratio of Risk-Stratified Screening for Breast Cancer: A Life-Table Model. JAMA Oncol. 2018 Nov 1;4(11):1504–1510. doi: 10.1001/jamaoncol.2018.1901 29978189 PMC6230256

[16] Marmot MG, Altman DG, Cameron DA, Dewar JA, Thompson SG, Wilcox M. The benefits and harms of breast cancer screening: an independent review. Br J Cancer. 2013 Jun 11;108(11):2205–40. doi: 10.1038/bjc.2013.177 23744281 PMC3693450

[17] Corrêa RS. Mammography: Infrastructure, coverage, quality and risk of radio-induced cancer in opportunistic screening in the state of Goiás. [Mamografia: Infraestrutura, cobertura, qualidade e risco do câncer radioinduzido em rastreamento oportunístico no estado de Goiás] [DSc thesis]. [Goiás (Brazil)]: Universidade Federal de Goiás; 2012. 164 p. Available at: https://repositorio.bc.ufg.br/tede/bitstream/tde/1534/1/Tese%20Rosangela%20da%20S%20Correa.pdf. (Accessed on February 22, 2021).

[18] Hendrick RE. Radiation doses and cancer risks from breast imaging studies. Radiology. 2010 Oct;257(1):246–53. doi: 10.1148/radiol.10100570 20736332

[19] Migowski A, Nadanovsky P, Vianna CMM. Estimation of overdiagnosis in mammographic screening: a critical assessment. Revista Brasileira de Cancerologia 2021; 2021; 67(2): e–151281. doi: 10.32635/2176-9745.RBC.2021v67n2.1281

[20] Gøtzsche PC; Jørgensen K. Screening for breast cancer with mammography. Cochrane Database Syst Rev. 2013 Jun 4;(6):CD001877. doi: 10.1002/14651858.CD001877.pub5 23737396 PMC6464778

[21] Baines CJ, To T, Miller AB. Revised estimates of overdiagnosis from the Canadian National Breast Screening Study. Prev Med. 2016;90:66–71. doi: 10.1016/j.ypmed.2016.06.033 27374944

[22] Tomazelli JG, Migowski A, Ribeiro CM, Assis M, Abreu DM. Assessment of actions for breast cancer early detection in Brazil using process indicators: a descriptive study with Sismama data, 2010–2011. Epidemiol Serv Saude. 2017 Jan-Mar;26(1):61–70. English, Portuguese. doi: 10.5123/S1679-49742017000100007 27901188

[23] Fang F, Fall K, Mittleman MA, Sparén P, Ye W, Adami HO, et al. Suicide and cardiovascular death after a cancer diagnosis. N Engl J Med. 2012;366(14):1310–1318. doi: 10.1056/NEJMoa1110307 22475594

[24] Taylor C, Correa C, Duane FK et al. Estimating the Risks of Breast Cancer Radiotherapy: Evidence From Modern Radiation Doses to the Lungs and Heart and From Previous Randomized Trials. J Clin Oncol. 2017;35(15):1641–1649. doi: 10.1200/JCO.2016.72.0722 28319436 PMC5548226

[25] Clarke M, Collins R, Darby S, et al. Effects of radiotherapy and of differences in the extent of surgery for early breast cancer on local recurrence and 15-year survival: an overview of the randomised trials. Lancet. 2005 Dec 17;366(9503):2087–106. doi: 10.1016/S0140-6736(05)67887-7 . 16360786

[26] Sheth GR, Cranmer LD, Smith BD, Grasso-Lebeau L, Lang JE. Radiation-Induced Sarcoma of the Breast: A Systematic Review. Oncologist. 2012;17(3):405–18. doi: 10.1634/theoncologist.2011-0282 22334455 PMC3316927

[27] Gervais MK, Burtenshaw SM, Maxwell J, et al. Clinical outcomes in breast angiosarcoma patients: A rare tumor with unique challenges. J Surg Oncol. 2017 Dec;116(8):1056–1061. doi: 10.1002/jso.24780 29205355

[28] Torres KE, Ravi V, Kin K, et al. Long-term outcomes in patients with radiation-associated angiosarcomas of the breast following surgery and radiotherapy for breast cancer. Ann Surg Oncol. 2013;20(4):1267–74. doi: 10.1245/s10434-012-2755-y 23224828 PMC5036516

[29] Kerlikowske K, Zhu W, Hubbard RA, Geller B, Dittus K, Braithwaite D, et al. Outcomes of screening mammography by frequency, breast density, and postmenopausal hormone therapy. JAMA Intern Med. 2013;173(9):807–16. doi: 10.1001/jamainternmed.2013.307 23552817 PMC3699693

[30] Yaffe MJ, Mainprize JG. Risk of radiation-induced breast cancer from mammographic screening. Radiology. 2011;258(1):98–105. doi: 10.1148/radiol.10100655 21081671

[31] Fitzpatrick-Lewis D, Hodgson N, Ciliska D, Peirson P, Gauld M, Liu YY. Breast Cancer Screening. McMaster University, Hamilton, Ontario, Canada. Oct 2011. Available at: https://canadiantaskforce.ca/wp-content/uploads/2011/11/2011-breast-cancer-systematic-review-en.pdf. (Accessed on February 23, 2021).

[32] Myers ER, Moorman P, Gierisch JM, Havrilesky LJ, Grimm LJ, Ghate S, et al. Benefits and Harms of Breast Cancer Screening: A Systematic Review. JAMA. 2015 Oct 20;314(15):1615–34. doi: 10.1001/jama.2015.13183 26501537

[33] Da Costa Vieira RA, Biller G, Uemura G, Ruiz CA, Curado MP. Breast cancer screening in developing countries. Clinics. 2017;72(4):244–253. doi: 10.6061/clinics/2017(04)09 28492725 PMC5401614

[34] Ginsberg GM, Lauer JA, Zelle S, Baeten S, Baltussen R. Cost effectiveness of strategies to combat breast, cervical, and colorectal cancer in sub-Saharan Africa and South East Asia: mathematical modelling study. The BMJ. 2012;344:e614. doi: 10.1136/bmj.e614 22389347 PMC3292522

[35] Bretthauer M, Wieszczy P, Løberg M, Kaminski MF, Werner TF, Helsingen LM, et al. Estimated Lifetime Gained With Cancer Screening Tests: A Meta-Analysis of Randomized Clinical Trials. JAMA Intern Med. 2023 Aug 28:e233798. doi: 10.1001/jamainternmed.2023.3798 37639247 PMC10463170

[36] Oeffinger KC, Fontham ETH, Etzioni R, Herzig A, Michaelson JS, Shyh Y-C T, et al. Breast cancer screening for women at average risk: 2015 update from the American Cancer Society. JAMA. 2015; 314:1599–614. 26501536 10.1001/jama.2015.12783 PMC4831582

[37] Nelson HD, Fu R, Cantor A, Pappas M, Daeges M, Humphrey L. Effectiveness of Breast Cancer Screening: Systematic Review and Meta-analysis to Update the 2009 U.S. Preventive Services Task Force Recommendation. Ann Intern Med. 2016;164(4):244–55. doi: 10.7326/M15-0969 26756588

[38] Kim M, Kim H, Choi H, Son M, Lee KS, Han TH, et al. Age-Standardized Breast Cancer Detection Rates of Breast Cancer Screening Program by Age Group in Korea; Comparison with Age-Standardized Incidence Rates from the Korea Central Cancer Registry. Healthcare (Basel). 2020 May 11;8(2):132. doi: 10.3390/healthcare8020132 32403445 PMC7349209

[39] de Camargo Cancela M, de Oliveira Santos M, Migowski A, Piñeros M. Breast cancer among young women in Brazil: Differences between hospital and population-based series. Cancer Epidemiol. 2022 Aug;79:102193. doi: 10.1016/j.canep.2022.102193 35696767

[40] van Ravesteyn NT, Schechter CB, Hampton JM, Alagoz O, van den Broek JJ, Kerlikowske K, et al. Trade-Offs Between Harms and Benefits of Different Breast Cancer Screening Intervals Among Low-Risk Women. J Natl Cancer Inst. 2021 Jan 30:djaa218. doi: 10.1093/jnci/djaa218 33515225 PMC8502479

[41] Woloshin S, Jørgensen KJ, Hwang S, Welch HG. The New USPSTF Mammography Recommendations—A Dissenting View. N Engl J Med. 2023 Sep 16. doi: 10.1056/NEJMp2307229 37721382

[42] Qaseem A, Lin JS, Mustafa RA, Horwitch CA, Wilt TJ; Clinical Guidelines Committee of the American College of Physicians. Screening for Breast Cancer in Average-Risk Women: A Guidance Statement From the American College of Physicians. Ann Intern Med. 2019;170(8):547–560. doi: 10.7326/M18-2147 30959525

[43] Duffy SW, Vulkan D, Cuckle H, Parmar D, Sheikh S, Smith RA, et al. Effect of mammographic screening from age 40 years on breast cancer mortality (UK Age trial): final results of a randomised, controlled trial. Lancet Oncol. 2020 Sep;21(9):1165–1172. doi: 10.1016/S1470-2045(20)30398-3 32800099 PMC7491203

[44] Autier P. Breast cancer: Doubtful health benefit of screening from 40 years of age. Nat Rev Clin Oncol. 2015 Oct;12(10):570–2. doi: 10.1038/nrclinonc.2015.162 26370604

[45] Richman IB, Long JB, Soulos PR, Wang SY, Gross CP. Estimating Breast Cancer Overdiagnosis After Screening Mammography Among Older Women in the United States. Ann Intern Med. 2023 Aug 8. doi: 10.7326/M23-0133 37549389 PMC10623662

[46] Brodersen J, Siersma VD. Long-term psychosocial consequences of false-positive screening mammography. Ann Fam Med. 2013;11(2):106–15. doi: 10.1370/afm.1466 ; PMCID: PMC3601385. 23508596 PMC3601385

[47] Hubbard RA, Kerlikowske K, Flowers CI, Yankaskas BC, Zhu W, Miglioretti DL. Cumulative probability of false-positive recall or biopsy recommendation after 10 years of screening mammography: a cohort study. Ann Intern Med. 2011 Oct 18;155(8):481–92. doi: 10.7326/0003-4819-155-8-201110180-00004 22007042 PMC3209800

[48] Bruening W, Fontanarosa J, Tipton K, Treadwell JR, Launders J, Schoelles K. Systematic review: comparative effectiveness of core-needle and open surgical biopsy to diagnose breast lesions. Ann Intern Med. 2010;152(4):238–46. doi: 10.7326/0003-4819-152-1-201001050-00190 20008742

[49] Ho AY, Bellon JR. Overcoming Resistance—Omission of Radiotherapy for Low-Risk Breast Cancer. N Engl J Med. 2023;388(7):652–653. doi: 10.1056/NEJMe2216133 36791166

[50] Migowski A. Estimation of Breast Cancer Overdiagnosis in a U.S. Breast Screening Cohort. Ann Intern Med. 2022 Oct;175(10):W114. doi: 10.7326/L22-0273 36252251

[51] Zahl PH, Jørgensen KJ, Gøtzsche PC. Overestimated lead times in cancer screening has led to substantial underestimation of overdiagnosis. Br J Cancer. 2013;109(7):2014–9. doi: 10.1038/bjc.2013.427 23963144 PMC3790152

[52] Lehman CD, Arao RF, Sprague BL, Lee JM, Buist DS, Kerlikowske K, et al. National Performance Benchmarks for Modern Screening Digital Mammography: Update from the Breast Cancer Surveillance Consortium. Radiology. 2017;283(1):49–58. doi: 10.1148/radiol.2016161174 27918707 PMC5375631

[53] Grabler P, Sighoko D, Wang L, Allgood K, Ansell D. Recall and Cancer Detection Rates for Screening Mammography: Finding the Sweet Spot. AJR Am J Roentgenol. 2017;208(1):208–213. doi: 10.2214/AJR.15.15987 27680714

[54] Mattos JSC, Caleffi M, Vieira RAC. Breast cancer screening in Brazil: Preliminary results. [Rastreamento mamográfico no Brasil: Resultados preliminares]. Rev Bras Mastologia. 2013;23(1):22–27.

[55] Welch HG, Prorok PC, O’Malley AJ, Kramer BS. Breast-Cancer Tumor Size, Overdiagnosis, and Mammography Screening Effectiveness. N Engl J Med. 2016 Oct 13;375(15):1438–1447. doi: 10.1056/NEJMoa1600249 27732805

[56] Birnbaum J, Gadi VK, Markowitz E, Etzioni R. The Effect of Treatment Advances on the Mortality Results of Breast Cancer Screening Trials: A Microsimulation Model. Ann Intern Med. 2016 Feb 16;164(4):236–43. doi: 10.7326/M15-0754 26756332 PMC5356482

[57] Harkness EF, Astley SM, Evans DG. Risk-based breast cancer screening strategies in women. Best Pract Res Clin Obstet Gynaecol. 2020;65:3–17. doi: 10.1016/j.bpobgyn.2019.11.005 31848103

[58] Román M, Sala M, Domingo L, Posso M, Louro J, Castells X. Personalized breast cancer screening strategies: A systematic review and quality assessment. PLoS One. 2019 Dec 16;14(12):e0226352. doi: 10.1371/journal.pone.0226352 31841563 PMC6913984

[59] Ferlay J, Soerjomataram I, Dikshit R, Eser S, Mathers C, Rebelo M et al. Cancer incidence and mortality worldwide: sources, methods and major patterns in GLOBOCAN 2012. Int J Cancer. 2015;136(5):E359–86. doi: 10.1002/ijc.29210 25220842

[60] França E, Teixeira R, Ishitani L, et al. Ill-defined causes of death in Brazil: a redistribution method based on the investigation of such causes. Rev Saude Publica 2014;48(4):671–681. doi: 10.1590/s0034-8910.2014048005146 25210826 PMC4181094

[61] Couto MSA, Firme VAC, Guerra MR, Bustamante-Teixeira MT. The effect of redistribution of ill-defined causes of death on the mortality rate of breast cancer in Brazil. Cien Saude Colet. 2019 Sep 9;24(9):3517–3528. Portuguese, English. doi: 10.1590/1413-81232018249.31402017 31508769

[62] Hu X, Ma J, Jemal A, Zhao J, Nogueira L, Ji X, et al. Suicide Risk Among Individuals Diagnosed With Cancer in the US, 2000–2016. JAMA Netw Open. 2023 Jan 3;6(1):e2251863. doi: 10.1001/jamanetworkopen.2022.51863 36662522 PMC9860529

[63] Michalek IM, Caetano Dos Santos FL, Wojciechowska U, Didkowska J. Suicide after a Diagnosis of Cancer: Follow-Up of 1.4 Million Individuals, 2009–2019. Cancers (Basel). 2023 Aug 29;15(17):4315. doi: 10.3390/cancers15174315 37686591 PMC10486959

[64] Kazlauskiene J, Navickas A, Lesinskiene S, Bulotiene G. Factors Affecting Suicidal Thoughts in Breast Cancer Patients. Medicina (Kaunas). 2022 Jun 28;58(7):863. doi: 10.3390/medicina58070863 35888582 PMC9322153

[65] Racadot S, Arnaud A, Schiffler C, Metzger S, Pérol D, Kirova Y. Cicaderma® in radiation-related dermatitis of breast cancer: Results from the multicentric randomised phase III CICA-RT. Clin Transl Radiat Oncol. 2023 Jun 1;41:100647. doi: 10.1016/j.ctro.2023.100647 37441546 PMC10334129

[66] Bontempo PSM, Ciol MA, Menêses AG, Simino GPR, Ferreira EB, Reis PEDD. Acute radiodermatitis in cancer patients: incidence and severity estimates. Rev Esc Enferm USP. 2021 Apr 16;55:e03676. doi: 10.1590/S1980-220X2019021703676 33886907

