# Supplementary material for: Harms and benefits of mammographic screening for breast cancer in Brazil
Source: PLoS One. 2024 Jan 25;19(1):e0297048. doi: 10.1371/journal.pone.0297048 (PMC10810469; doi:10.1371/journal.pone.0297048)
Supplement: S1 File — (DOCX) [file pone.0297048.s001.docx]

**Supporting information**

**Detailed explanations of each scenario**

The baseline value of breast cancer mortality, mortality due to possible harms and cancer detection rate were maintained in the three scenarios. The relative benefit and harm risks that were applied to the Brazilian population in the estimation of the three possible scenarios varied, as follows:

a) Best case scenario: scenario most favorable to mammographic screening, i.e. increase in deaths avoided and reduction in deaths caused in comparison with the most likely scenario.

b) Base case: most likely scenario according to the best available evidence for benefits and harms as described in table 1S.

c) Worst case scenario: most unfavorable scenario for mammographic screening, i.e. reduction in deaths avoided and increase in deaths caused by screening (direct or indirectly), when compared to the base case.

In general, point estimates were used for the base case (most likely scenario based on the best available evidence), and lower and upper limits of 95% confidence interval estimates were used for the other two scenarios (Tables 2S and 3S). For each screening protocol, these three scenarios for screening harms and three scenarios for benefits were simulated, totaling nine possible combinations of balances between benefits and harms for each protocol. In addition to these nine scenarios for each protocol, additional sensitivity analyses were performed for benefits and harms (Tables 2S, 3S, 4S and 5S).

Benefits of annual screening for 40 to 49 years old: in the best-case scenario, RR was from a meta-analysis of randomized controlled trials on mammographic screening effect on breast cancer mortality (AHRQ Publication No. 23-05303-EF-1. 2023, available at: https://www.uspreventiveservicestaskforce.org/ uspstf/document/ draft-evidence-review/breast-cancer-screening-adults), which did not reach statistical significance. Therefore, for the base case and worst-case scenarios screening was considered to have a null effect on mortality. Benefits of biennial screening for ages 50 to 59, 60 to 69 and 50 to 69: the RR from the same meta-analysis aforementioned was used for the base case, and the values of the lower and upper limits of the 95% confidence interval were used for the worst and best-case scenarios. Benefits of annual screening for ages 40 to 49, followed by biennial screening for ages 50 to 69 years: in the best scenario, for screening for 40 to 49 years, the RR value of the aforementioned meta-analysis and for the age group 50 to 69 years, the upper limit of the 95% confidence interval from this same meta-analysis was used for this age group.

In the sensitivity analysis of the harms, the estimates used for each of the scenarios were as follows:

Death from breast cancer induced by radiation from mammograms: In the best-case scenario, estimates based on Brazilian data for analog mammography were used. For the base case, estimates based on the same Brazilian study were used, only for CR digital mammography which is very common in the country. For the worst-case scenario, in addition to using the estimates for CR digital mammography machines, additional doses of radiation from the diagnostic investigation of false-positive cases in screening were also added, as detailed previously. Deaths by suicide associated with overdiagnosis: in the base case, the point estimate of the RR was used, with the 95%CI limits being used for the worst and best-case scenarios, respectively (table 3S). Cardiovascular deaths associated with overdiagnosis: in the base case, the point estimate of the RR was used, with the upper and lower limits of 95%CI being used for the worst and best-case scenarios, respectively (table 3S).

Surgical deaths associated with excess mastectomies were not included in the best-case scenario. Stewart-Treves syndrome was considered only in the worst-case scenario (table 3S). Cardiovascular deaths associated with adjuvant radiotherapy: the worst case scenario was based on the point estimate of increase in RR from the meta-analysis of adjuvant radiotherapy clinical trials was used (table 3S). These data available in the literature relate to old treatments due to the long period of observation necessary to quantify all harms, which may have involved higher radiation doses than those used in current radiotherapy regimens, hence its use in the worst-case scenario. For the base case and the best-case scenario, the RR estimates calculated for the present model were used for average doses used in modern radiotherapy according to the literature, as explained previously. Another difference is that for the base case and for the worst-case scenario, the same ICD 10 codes used in the original meta-analysis were used, while in the best-case scenario, fewer ICD 10 codes were used, selected especially for the sensitivity analyses according to the greater biological plausibility. A variation in the duration of the increased risk was also used for sensitivity analysis purposes. Therefore, the duration of the increase in risk was shorter in the best-case scenario than in base case and worst scenarios, in which the increase in risk began immediately after overtreatment and persisted for 20 years, as observed in the meta-analysis of clinical trials. In the best-case scenario, the increase in risk in the model began only in the fifth year after treatment and ended after 15 years.

Table 1S - Main sources of information for the outcomes included in the study.

| **Type of outcome** | **Outcome** | **Main sources** |
| --- | --- | --- |
| Screening benefit | Breast cancer mortality | National life tables and meta-analysis of screening clinical trials |
| Screening harms | Cumulative proportion of false positives | Screening prospective cohort |
|  | Cumulative proportion of excessive biopsies | Prospective cohort and screening clinical trials |
|  | Death from breast cancer radioinduced by mammograms | Simulation models of cancer induction by ionizing radiation |
|  | Deaths by Suicide | Population-based cohort |
|  | Cardiovascular death |  |
|  | Surgical mortality after mastectomy | Case series |
|  | Death from Stewart-Treves syndrome |  |
|  | Death from coronary artery disease associated with radiotherapy | Meta-analysis of clinical trials of localized breast cancer treatment |
|  | Death from breast cancer associated with radiotherapy |  |
|  | Death from lung cancer associated with radiotherapy |  |
|  | Death from esophageal cancer associated with radiotherapy |  |
|  | Death from pulmonary embolism associated with radiotherapy |  |

Source: own elaboration

Table 2S - Parameters from the literature used to estimate the benefits of screening.

**Age group**

**Relative risk**

**References**

40-49 years (best scenario)

0.92 (CI 95%: 0.75-1.02)

AHQR,2023

40-49 years (sensitivity analysis.)

0.93 (CI 95%: 0.80-1.09)

Moss, 2015

40-49 years (sensitivity analysis.)

0.88 (CI 95%: 0.73-1.003)

Nelson 2016a

50-59 years

0.86 (CI 95%: 0.68-0.97)

AHQR,2023

60-69 years

0.67 (CI 95%: 0.54-0.83)

AHQR,2023

50-69 years (sensitivity analysis.)

0.80 (CI 95%: 0.73-0.89)

Marmot, 2012

50-69 years

0.78 (CI 95%: 0.68-0.90)

AHQR,2023

70-74 years

0.80 (CI 95%: 0.51-1.28)

AHQR,2023

Legend: RR = Relative Risk; CI = Confidence Interval

Source: own elaboration

Agency for Healthcare Research and Quality. Screening for Breast Cancer: A Comparative Effectiveness Review for the U.S. Preventive Services Task Force. AHRQ 2023. AHRQ Publication No. 23-05303-EF-1. Available at: https://www.uspreventiveservicestaskforce.org/uspstf/document/draft-evidence-review/breast-cancer-screening-adults

Table 3S - Parameters from the literature used to estimate the harms of screening.

| **Variables** | **Parameters** | | | | | | | **References** | | | | | |
| --- | --- | --- | --- | --- | --- | --- | --- | --- | --- | --- | --- | --- | --- |
| Attendance rate | 80% | | | | Marmot et al, 2012 | | | | | | |  |  |
| Number of cases of radiation-induced  cancer by mammograms per 100,000 women 2.32 Corrêa, 2012  aged 50 - 69 years, screened biennially | | | | | | | | | |  |  |  |  |
|  |  | | | |  | | | | | | |  |  |
| Cumulative percentage of false positives* | Various, according to age group, periodicity of screening and breast density. | | | | Kerlikowske, 2013 | | | | | | |  |  |
| Cumulative percentage of excessive biopsies* |  |  |  |  |  |  |  |  |  |  |  |  |  |
| Cancer detection rate in the first screening round | |  | | | | | | | |  |  |  |  |
| At age 40 | 0.22% | | | | Mattos, 2013 | | | | | | |  |  |
| At age 50 | | | 0.55% | | | Mattos, 2013 | | | | |  |  |  |
| Percentage of overdiagnosis by age group | | |  | | |  | | | | |  |  |  |
| 40-49 years | | | 41% | | | | Baines et al, 2016 | | | | | |  |
| 50-59 years | | | 25% | | | |  |  |  |  |  |  |  |
| 60-69 years | | | 28% | | | | Welch, 2006 | | | | | |  |
| Percentage of cases in stages 0 and I treated surgically | | | 100% | | | | Sagara et al, 2015 | | | | | |  |
| Percentage of conservative surgery in stages 0 and I | | | 73% | | | | Ribeiro et al, 2013 | | | | | |  |
| Percentage of mastectomy in stages 0 and I | | | 27% | | | |  |  |  |  |  |  |  |
| Percentage of reoperation with mastectomy after conservative surgery | | | 7.70% | | | | Jeevan et al, 2012 | | | | | |  |
| Percentage of adjuvant radiotherapy after conservative surgeries | | | 100% | | | | Ribeiro et al, 2013 | | | | | |  |
| Surgical mortality after mastectomy | | | 0.24% | | | | El-Tamer, 2007 | | | | | |  |
| Percentage of post-mastectomy reconstruction | | | 70% | | | | Ribeiro et al, 2013 | | | | | |  |
| Stewart-Treves syndrome | | |  | | | |  | | | | | |  |
| Post-mastectomy incidence | | | 0,07% | | | | Berebichez-Fridman  et al, 2016 | | | | | |  |
| Five-year survival rate | | | 10% | | | | Wierzbicka-Hainaut  et al, 2010 | | | | | |  |
| RR of suicide after diagnosis of breast cancer | | | 3,4 (IC 95%: 1,3 - 6,9) | | | | Fang et al, 2012 | | | | | |  |
| RR of cardiovascular death after breast cancer diagnosis | | | 1,8 (IC 95%: 1,2 - 2,4) | | | |  |  |  |  |  |  |  |
| RR of lung cancer after adjuvant radiotherapy | | | 2,13 (IC 95%: 1,14 - 3,98) | | | | Taylor et al, 2017 | | | | | |  |
| RR of cardiovascular disease cancer after adjuvant radiotherapy | | | 1,3 (IC 95%: 1,15 - 1,46) | | | |  |  |  |  |  |  |  |
| RR of pulmonary thromboembolism after adjuvant radiotherapy | | | 2,1 (IC 95%: 1,11 - 3,90) | | | |  |  |  |  |  |  |  |
| RR of esophageal cancer cancer after adjuvant radiotherapy | | | 2,51 (IC 95%: 1,08 - 5,72) | | | |  |  |  |  |  |  |  |
| Additional incidence of breast sarcoma up to 15 years after adjuvant radiotherapy | | | 0,09% | | | | Sheth GR et al, 2012 | | | | | |  |
| 5-year survival of radiation-induced breast sarcoma | | | 27% | | | |  |  |  |  |  |  |  |
| Source: own elaboration | | |  |  | | | | |  |  |  |  |  |

Table 4S - Number of deaths due to radiation-induced breast cancer, by attendance rate (100% or 80%), type of mammogram and screening protocol.

| **Screening protocol** | **Number of deaths from radiation-induced breast cancer by number of**  **women screened and type of mammogram** | | | |
| --- | --- | --- | --- | --- |
|  | **CR mammography** | | **Analog mammography** | |
|  | **10,000** | **8,000** | **10,000** | **8,000** |
| 40-49, annual | 1.05 | 0.84 | 0.68 | 0.54 |
| 50-59, biennial | 0.16 | 0.13 | 0.10 | 0.08 |
| 60-69, biennial | 0.07 | 0.06 | 0.05 | 0.04 |
| 50-69, biennial | 0.23 | 0.19 | 0.15 | 0.12 |
| 40-49, annual + 50-69, biennial | 1.28 | 1.03 | 0.83 | 0.66 |
| Source: own elaboration. Legend: CR = Computed Radiography | |  | | |

Table 5S - Number of deaths due to radiation-induced cancer per number of women screened, type of mammogram and screening protocol, considering the additional radiation from the diagnostic investigation of false-positive cases.

| **Screening protocol** | **Number of deaths from radiation-induced breast cancer by number of**  **women screened and type of mammogram** | | | |
| --- | --- | --- | --- | --- |
|  | **CR mammography** | | **Analog mammography** | |
|  | **10000** | **8000** | **10000** | **8000** |
| 40-49, annual | 1.16 | 0.93 | 0.75 | 0.60 |
| 50-69, biennial | 0.26 | 0.20 | 0.17 | 0.13 |
| 40-49, annual + 50-69, biennial | 1.41 | 1.13 | 0.91 | 0.73 |
|  |  |  |  |  |
| Legend: CR = Computed Radiography. | | |  |  |
| Source: own elaboration | |  |  |  |

Table 6S – Cancer detection rates in mammographic screening in Brazilian studies.

| **Age group** | **Cancer detection rate by municipality studied** | | | |
| --- | --- | --- | --- | --- |
|  | **Barretos, SP^1^** | **Campinas, SP^2^** | **Monteiro, PB^3^** | **São Paulo, SP^4^** |
| 40-44 | 0.224 | 0.2 | NA | NA |
| 45-49 | 0.514 | 0.2 | NA | NA |
| 50-59 | 0.548 | 0.2 | NA | NA |
| 60-69 | 0.805 | 0.6 | NA | NA |
| Total | 0.5 | 0.34 | 0.34 | 0.324 |

Source: own elaboration. Legend: NA = Not Available; SP = São Paulo; PB = Paraíba.. References: ^1.^Mattos JSC, Caleffi M, Vieira RAC. [Rastreamento mamográfico no Brasil: Resultados preliminares]. Rev Bras Mastologia. 2013;23(1):22-27 ; ^2.^ Camargo Júnior HS, Camargo MM, Teixeira SR, Arruda MS, Azevedo J. [Apresentação de resultados de um serviço de rastreamento mamográfico com ênfase na auditoria epidemiológica]. Rev Bras Ginecol Obstet. 2009 Oct;31(10):508-12.; ^3.^Barreto AS, Mendes MF, Thuler LC. [Avaliação de uma estratégia para ampliar a adesão ao rastreamento do câncer de mama no Nordeste brasileiro]. Rev Bras Ginecol Obstet. 2012;34(2):86-91. ; ^4.^ Caetano S, Junior JM, Finguerman F, Goldman SM, Szejnfeld J. [Mammographic assessment of a geographically defined population at a mastology referral hospital in São Paulo Brazil]. PLoS One. 2013 Sep 16;8(9):e74270. doi: 10.1371/journal.pone.0074270.

Tabela 7S. Cancer detection rates by detailed age group estimated for Brazil for screening from 40 to 69 years of age compared to those available for the USA.

| **Age group** | **CDR (%)** | | | |
| --- | --- | --- | --- | --- |
|  | **Brazil** | **BCSC (USA)** | | |
| 40 years* | 0.224 | NA | | |
| 41-44 years | 0.178 | 0.265 | | |
| 45-49 years | 0.258 | 0.364 | | |
| 50-54 years | 0.330 | 0.428 | | |
| 55-59 years | 0.438 | 0.467 | | |
| 60-64 years | 0.557 | 0.570 | | |
| 65-69 years | 0.675 | 0.612 | | |
| *first screening round. From the age of 41 onwards, the parameters are for subsequent screening rounds. Source: own elaboration. Legend: CDR: Cancer Detection Rate; BCSC: Breast Cancer Screening Consortium; NA: Not Available. BCSC data available at: http://www.bcsc-research.org/statistics/performance/ screening/2009/rate_age.html. | | | |  |
|  | | |  |  |

**Comparison of the benefits of screening in the present study with other results in the literature**

The absolute benefit of biennial screening from 50 to 69 years of age estimated for Brazil was similar to that observed in clinical trials of mammography screening (Table 9S), but was about half of the estimate of the effect of screening in the United Kingdom in the same decade (Table 8S).

Table 8S – Comparison of the effectiveness of mammographic screening between Brazil and the United Kingdom

40-49 annual

United Kingdom

Brazil

Brazil

Relative risk (RR)

80%

80%

93%

Women invited to screen (n)

10,000

10,000

10,000

Breast cancer deaths prevented (n)

43

21

3

NNS

235

481

3,387

Screening effectiveness outcomes

Efetividade do rastreamento

50-69 years triennial / biennial

Legend: NNS = Number needed to be invited to screen to avoid one breast cancer death.

*Source (for the United Kingdom): Marmot MG et al. The benefits and harms of breast cancer screening: an independent review. A report jointly commissioned by Cancer Research UK and the Department of Health (England). London, 2012. Source (for Brazil): own elaboration.

Table 9S – Comparison of the effectiveness of mammographic screening between Brazil and randomized clinical trials, 10,000 women for 10 years.

| Age group and  relative risk* | Number of breast cancer deaths prevented by screening | |
| --- | --- | --- |
|  | Brazil | Clinical trials* |
| 40-49 years, 0.92 | 3.4 | 2.9 |
| 50-59 years, 0.86 | 8.3 | 7.7 |
| 60-69 years, 0.67 | 23.0 | 21.3 |
| 70-74 years, 0.80 | 11.0 | 13.0 |
| Source: own elaboration. |  |  |

*Nelson HD, Fu R, Cantor A, Pappas M, Daeges M, Humphrey L. Effectiveness of Breast Cancer Screening: Systematic Review and Meta-analysis to Update the 2009 U.S. Preventive Services Task Force Recommendation. Ann Intern Med. 2016b;164(4):244-55. doi: 10.7326/M15-0969.

Table 10S – Benefits and harms of mammographic screening in the base case, for 10,000 women invited for mammography screening over 10 years, according to the age group and screening interval.

| **Screening interval** | | **Annual** | **Biennial** | |
| --- | --- | --- | --- | --- |
| **Age group** | | **40 - 49 years** | **50 – 59 years** | **60 – 69 years** |
| Benefits | Breast cancer deaths prevented (n) | 0 | 8 | 23 |
| Harms | False positives (n) | 5088 | 2659 | 2515 |
|  | Excessive biopsies (n) | 871 | 409 | 383 |
|  | Overdiagnosis (n) | 72 | 41 | 61 |
|  | Overtreated with mastectomy (n) | 19 | 11 | 17 |
|  | Overtreated with radiotherapy (n) | 49 | 28 | 41 |

Source: own elaboration.

Table 11S – Benefits and harms of mammographic screening in the base case, for 2,000 women invited for mammography screening over 10 years, according to the age group and screening interval.

| **Screening interval** | | **annual** | **biennial** | |
| --- | --- | --- | --- | --- |
| **Faixa etária** | | **40 - 49 years** | **50 – 59 years** | **60 – 69 years** |
| Benefits | Breast cancer deaths prevented (n) | 0 | 2 | 5 |
| Harms | False positives (n) | 1018 | 532 | 503 |
|  | Excessive biopsies (n) | 174 | 82 | 77 |
|  | Overdiagnosis (n) | 14 | 8 | 12 |
|  | Overtreated with mastectomy (n) | 5 | 3 | 4 |
|  | Overtreated with radiotherapy (n) | 10 | 6 | 8 |

Source: own elaboration.

**Additional results to those presented in the article**

Table 12S - Percentage of causes of death associated with annual screening in those aged 40 to 49 years, followed by biennial screening from 50 to 69 years.

| Cause of death | Best scenario | Base case | Worst scenario |
| --- | --- | --- | --- |
| Breast Cancer Radioinduced by Mammograms | 44.40 | 36.80 | 26.49 |
| Overtreatment with Surgery | 0.00 | 6.18 | 4.98 |
| Overtreatment with Radiotherapy (cardiovascular diseases) | 36.50 | 46.31 | 48.58 |
| Overtreatment with Radiotherapy (lung cancer) | 18.84 | 10.10 | 11.94 |
| Overtreatment with Radiotherapy (esophageal cancer) | 0.00 | 0.00 | 2.81 |
| Overtreatment with Radiotherapy (pulmonary embolism) | 0.00 | 0.00 | 2.88 |
| Overtreatment with Radiotherapy (sarcomas) | 0.00 | 0.00 | 1.81 |
| Overdiagnosis (suicide) | 0.03 | 0.11 | 0.18 |
| Overdiagnosis (cardiovascular disease) | 0.23 | 0.50 | 0.33 |
| Source: own elaboration. |  |  |  |

Table 13S - Percentages of causes of death associated with biennial screening in those aged 50 to 59 years.

| Cause of death | Best scenario | Base case | Worst scenario |
| --- | --- | --- | --- |
| Breast Cancer Radioinduced by Mammograms | 31.65 | 25.43 | 17.03 |
| Overtreatment with Surgery | 0.00 | 8.01 | 6.00 |
| Overtreatment with Radiotherapy (cardiovascular diseases) | 39.29 | 50.83 | 51.58 |
| Overtreatment with Radiotherapy (lung cancer) | 28.71 | 14.91 | 16.36 |
| Overtreatment with Radiotherapy (esophageal cancer) | 0.00 | 0.00 | 3.42 |
| Overtreatment with Radiotherapy (pulmonary embolism) | 0.00 | 0.00 | 2.77 |
| Overtreatment with Radiotherapy (sarcomas) | 0.00 | 0.00 | 2.18 |
| Overdiagnosis (suicide) | 0.04 | 0.17 | 0.26 |
| Overdiagnosis (cardiovascular disease) | 0.31 | 0.65 | 0.40 |
| Source: own elaboration. |  |  |  |

Table 14S - Percentages of causes of death associated with biennial screening in those aged 60 to 69 years.

| Cause of death | Best scenario | Base case | Worst scenario |
| --- | --- | --- | --- |
| Breast Cancer Radioinduced by Mammograms | 4.26 | 3.08 | 1.95 |
| Overtreatment with Surgery | 0.00 | 3.27 | 2.32 |
| Overtreatment with Radiotherapy (cardiovascular diseases) | 77.60 | 84.14 | 80.55 |
| Overtreatment with Radiotherapy (lung cancer) | 17.46 | 8.18 | 8.47 |
| Overtreatment with Radiotherapy (esophageal cancer) | 0.00 | 0.00 | 2.27 |
| Overtreatment with Radiotherapy (pulmonary embolism) | 0.00 | 0.00 | 2.77 |
| Overtreatment with Radiotherapy (sarcomas) | 0.00 | 0.00 | 0.84 |
| Overdiagnosis (suicide) | 0.02 | 0.09 | 0.13 |
| Overdiagnosis (cardiovascular disease) | 0.66 | 1.23 | 0.71 |
| Source: own elaboration. |  |  |  |

# Table 15S - Estimates of harms of overtreatment with mastectomy for 10,000 women invited for mammographic screening in a 10-year period in Brazil.

| **Type of harm associated with**  **overtreatment with surgery** | **Screening protocol** | | |
| --- | --- | --- | --- |
|  | **40-49 years, annual** | **50-59 years, biennial** | **60-69 years, biennial** |
| Number of cases initially overtreated  with mastectomy | 19.482 | 11.057 | 16.562 |
| Number of cases with conservative surgery who undergo reoperation with mastectomy | 4.056 | 2.302 | 3.448 |
| Number of deaths in cases with reconstruction (considering 70% of mastectomized patients) | 0.054 | 0.031 | 0.046 |
| Number of deaths in cases without reconstruction | 0.017 | 0.010 | 0.014 |
| Total number of surgical deaths | 0.071 | 0.040 | 0.061 |
| Number of deaths from Stewart-Treves syndrome (worst case scenario only) | 0.016 | 0.009 | 0.014 |

Source: own elaboration.
